# Neural Correlates of Social Behavior in Mushroom Body Extrinsic Neurons of the Honeybee *Apis mellifera*

**DOI:** 10.3389/fnbeh.2020.00062

**Published:** 2020-04-21

**Authors:** Benjamin H. Paffhausen, Inga Fuchs, Aron Duer, Isabella Hillmer, Ioanna M. Dimitriou, Randolf Menzel

**Affiliations:** Neurobiology, Institute of Biology, Freie Universität Berlin, Berlin, Germany

**Keywords:** social interactions, extracellular recordings, mini colony, motivated behavior, mushroom body, high order interactions

## Abstract

The social behavior of honeybees (*Apis mellifera*) has been extensively investigated, but little is known about its neuronal correlates. We developed a method that allowed us to record extracellularly from mushroom body extrinsic neurons (MB ENs) in a freely moving bee within a small but functioning mini colony of approximately 1,000 bees. This study aimed to correlate the neuronal activity of multimodal high-order MB ENs with social behavior in a close to natural setting. The behavior of all bees in the colony was video recorded. The behavior of the recorded animal was compared with other hive mates and no significant differences were found. Changes in the spike rate appeared before, during or after social interactions. The time window of the strongest effect on spike rate changes ranged from 1 s to 2 s before and after the interaction, depending on the individual animal and recorded neuron. The highest spike rates occurred when the experimental animal was situated close to a hive mate. The variance of the spike rates was analyzed as a proxy for high order multi-unit processing. Comparing randomly selected time windows with those in which the recorded animal performed social interactions showed a significantly increased spike rate variance during social interactions. The experimental set-up employed for this study offers a powerful opportunity to correlate neuronal activity with intrinsically motivated behavior of socially interacting animals. We conclude that the recorded MB ENs are potentially involved in initiating and controlling social interactions in honeybees.

## Introduction

Social behavior requires the integration of multisensory inputs, evaluation of their meaning in the social context and their relevance for the current needs and aims of the animal. Perception, evaluation, internal status probing, and decision making will have to interact on the neural level. Multiple studies predominantly in laboratory mammals have established endocrine and global brain centers (e.g., in mammals the amygdala, the prefrontal cortex) that could be correlated with aggression, mating, social bonding, affiliate behavior and other aspects of social behavior (Insel, 1997; Adolphs, 2001; Lim and Young, 2006; Rilling et al., 2008; Stanley and Adolphs, 2013). However, the organization of small central neural networks or even single neurons in the control of social behavior is unknown. We would expect convergent neurons to be involved in high order sensory coding, in the evaluation of their meaning for the animal in its current status and participation in social actions. Social and pre-social insects might offer model systems for analyzing such neural networks (Choe and Crespi, 1997; Farris, 2016). The fly *Drosophila melanogaster*, for example, improves long-term memory retrieval in a social context (Chabaud et al., 2009) indicating that even in a pre-social insect, group interactions affect experience-dependent behavior, and neural correlates can be discovered. The circuits involved are anatomically and functionally related to the mushroom body (MB), a high order integration and memory storage device.

Eusocial insects like the honeybee are characterized by cooperative brood care, overlapping generations and age-dependent division of labor (Crespi and Yanega, 1995). The latter leads to a sequence of duties within the colony (attending the queen, feeding the larvae, cleaning and defending the colony) and finally outdoor behavior (exploring the environment, food foraging, searching and advertising for a new nest site; Rösch, 1925; Lindauer, 1954; Seeley, 1986). At any time, roughly a third of the workers feed and attends the queen and cares for the brood (nurse bees), another third cleans the hive, builds the comb and processes food (house bees) and the remaining colony members forage for pollen, nectar, resin and water (von Frisch, 1967; Robinson, 1992; Seeley, 1995). Bees communicate with chemical and mechanosensory signals about their social status, the conditions within the colony and those outside (food, water, resin, nest site; Farina, 1996). These social interactions are tightly connected with antennal probing of other colony members, the wax surface, and the larvae (Farina et al., 2005). Social interactions can even act as appetitive reinforcers (Cholé et al., 2019). The tight net of social interactions qualifies the bee colony as an organism in itself, a superorganism, transcending the summed actions of its members (Seeley and Levien, 1987; Seeley, 2003). The elements of collective intelligence (Marshall and Franks, 2009), and thus the “wisdom of the hive” (Seeley, 1995) are the individual bees, whose perceptual and neural integrative properties define the underlying processes. A search for the neural correlates of collective behavior must, therefore, begin with a search in the brain of a bee actively participating in the social communication processes of a functional colony.

**Figure 1:**
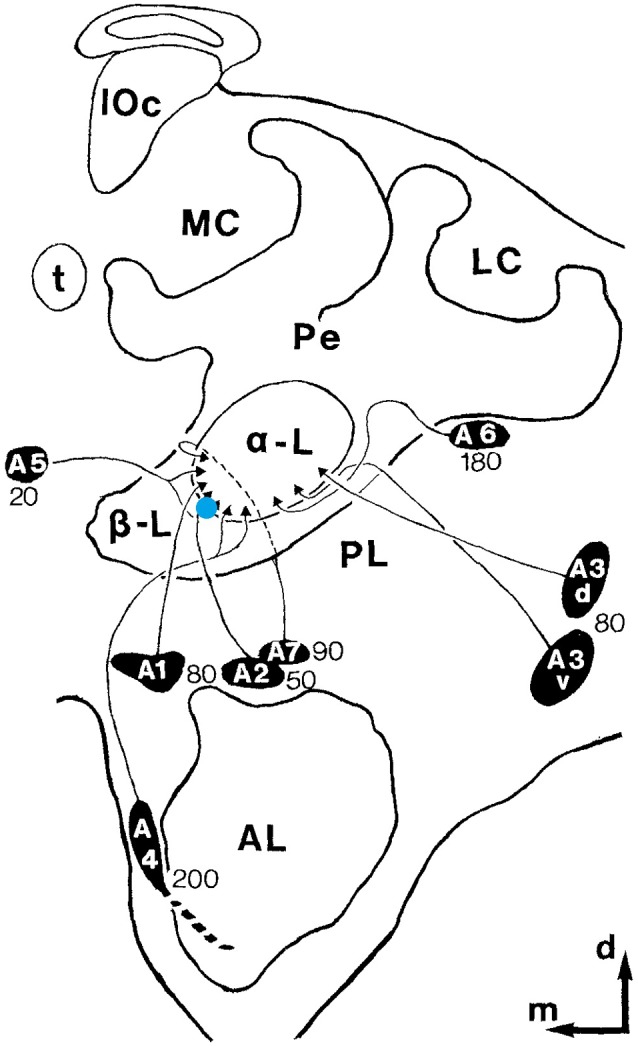
Recording site. Schematic drawing of one hemisphere of the honeybee brain. The blue dot depicts the recording site. MB ENs (mushroom body extrinsic neurons)of the alpha-lobe A1, A2, and A5 were targeted. The black ovals with the names of the neuron clusters mark the respective somata regions. The depth of theses neurons is indicated by the numbers near the clusters. The outer edge of the alpha-lobe can be recognized very well as a guide for electrode placement (adopted from Rybak and Menzel, 1993; Figure 3).

We focused our search on mushroom body extrinsic neurons (MB ENs) since the MB is known to be a high order, multimodal integration center in insects in general [honeybee: (Menzel et al., 1996), Drosophila: (Heisenberg, 1998), cockroach: (Mizunami et al., 2004; Rybak et al., 2010)]. MB ENs are well characterized anatomically, and were examined intensively in the context of olfactory learning and memory processing (Menzel, 2014). ENs of the alpha lobe project to multiple parts of the bee brain (Rybak and Menzel, 1993). They are multimodal (Erber, 1978; Homberg and Erber, 1979), and their responses are not related to motor activity (Mauelshagen, 1993; Rybak and Menzel, 1998) but to pre-motor descending pathways, and sensory processing neuropils (Rybak and Menzel, 1993). The MB also receives input from dopaminergic and octopaminergic value coding neurons, making the MB to a central place of neural plasticity, learning and memory processing (Aso et al., 2014). ENs include recurrent pathways to its input, the calyx, providing neural signals for identifying novel and already learned stimuli. These recurrent neurons are also thought to be involved in context-dependent forms of learning leading to attention dependent sensory processing and behavioral control (Filla and Menzel, 2015). Furthermore, ENs are known to change their response properties during olfactory classical conditioning (Strube-Bloss et al., 2011, 2016; Menzel, 2014) and instrumental visual learning (Zwaka et al., 2019). ENs of the classes A1, A2 and A5 are particularly interesting in our context because they change their activity during olfactory learning selectively for the learned stimulus (Okada et al., 2007; Strube-Bloss et al., 2011, 2016). A single identified neuron, the PE1 neuron, belongs to this group of ENs and was also found to undergo activity changes during olfactory learning (Mauelshagen, 1993). These A1, A2 and A5 ENs can be targeted by an extracellular electrode quite well because their integrating segments are located at the ventral-median margin of the alpha lobe (Figure 1), a site that is well recognized when exposing the brain for recordings. Furthermore, these less than 500 neurons exit the alpha lobe at a depth between 20–80 μm below the brain surface (Rybak and Menzel, 1993) making it easier to define the recording site. A different group of ENs, the A3 neurons exiting the alpha lobe at the median lateral margin, were recently successfully studied in honeybees that actively walked on a floating ball in a virtual reality setting to search for neural correlates of operant learning and memory retrieval (Zwaka et al., 2019). We previously developed a method that allowed us to record from ENs extracellularly in such a way that the animal was free to move within an area of 55 × 55 cm (Duer et al., 2015). Here we applied this method to search for neural correlates of social interactions within a small but functioning honeybee colony. Our method does not allow us to identify the particular neuron recorded within or between the A1, A2 and A5 groups making it very difficult to compare recordings across animals. We thus focused on analyses separately for each animal and afterward compare their properties across animals.

It has been proposed that the readout of the MB *via* ENs serves high order integration including context-dependence of learned behavior, expectation, and evaluation about learning events, social communication, navigation within the colony and outside and learning during symbolic forms of social interactions e.g., in the waggle dance (Menzel, 2012). Here we focused on social interactions inside the hive asking whether the recorded ENs were involved in social contacts and the initiation of social behavior. We found the neural correlates of several self-initiated behaviors in the social context.

## Materials and Methods

A detailed description of the experimental setup and procedures can be found in Duer et al. (2015). The experimental setup consisted of a honeybee colony housed in a Faraday cage and an electrophysiology recording station close by allowing to dissect an animal of the colony, implant electrodes, check the recording quality and move it with the recording electrode into the experimental hive. The floor of the hive (55 cm × 55 cm) was made of sheets of beeswax. It was tilted by 17° to the horizontal. Since bees are highly sensitive to gravity (Markl, 1974) they could use gravity for orienting in the hive. We observed normal dance behavior with waggles runs oriented to gravity. The support under the floor contained heating elements keeping it at 32–34°C. A barrier between the bee wax layer and the heating elements was made of a 1 cm thick metal plate to stabilize the temperature and shield any electric fields from the heating circuit and other devices. A 10 cm high plastic board sprayed with dry silicone PTFE prevented bees from climbing up the walls and leaving the hive. A hole in the plastic board was connected to a tube that connected the hive to the outside world. Foraging bees shuttled regularly between inside and outside. A hood covered the hive from light, temperature fluctuations, wind drafts, and electric noise. The hood consisted of wood and a metal mesh. The head stages of the AC amplifier (EXT, npi electronics, Tamm, Germany), the infra-red LEDs and a webcam (Logitech Pro 9000, Logitech international S.A., Apples, Switzerland, infrared filter removed) were arranged in a shielded box on top of the hood. The bottom side of the box pointing toward the arena had a DIP plug installed where the electrodes were connected to the head stages.

The colony was started with about 1,000 young bees, brood and a queen in spring. Two weeks later the bees had built a brood nest and started storing nectar and pollen in close vicinity to the brood. If necessary young bees were added to the colony. Social communication, brood care, queen attending and food processing appeared normal throughout the time of experiments. An experiment started by selecting a bee from the colony that was not found to be close to the brood and did not leave the colony during a period of a few days before. Bees close to the brood might be nurses that might walk into the dense bee cluster surrounding the queen, a condition we wanted to avoid because bees could not be tracked with our video system in the dense cluster of bees there. Bees involved in foraging were also not suitable because they might tend to leave the hive. The selected bee was moved into a glass vial that was quickly transferred onto crushed ice. Dissection was performed under cold anesthesia by a stream of cold air. The temperature and airspeed was adjusted such that the antennae of the bee moved slowly but did not stop moving. Since the mechanoreceptors in the neck are very sensitive we avoided fixing the head with support in the neck and rather pinched the sturdy mandibles by tweezers that were attached to a micromanipulator. A Bowden mechanism allowed us to slowly close the tweezers, and the micromanipulator was used to position the tweezers around the mandibles with precision. A small window was cut into the head capsule above the alpha lobe. The trachea sack and glands were carefully pushed aside until the alpha lobe could be seen. The tip of the electrode bundle was attached to a micromanipulator and immediately placed at the edge of the alpha lobe (Figure 1). At this point, the bee was warmed up by switching off the cold air stream and by pouring 50°C hot water through thin tubing tightly surrounding the metal tube. This way the bee’s body temperature was raised to 35°C within a few seconds. Once the promising neuronal activity was observed the experiment was paused for 5 min to check for recording stability. If the spike shape and baseline of the signal were stable the opening in the head capsule was filled with two component silicone glue (KWIK-SIL, WPI, Sarasota, FL, USA). The heating was actively continued to this point and 5 min longer to make sure the silicone acted as a mechanical support. The silicone also prevented the brain from drying and moving with ventilation.

The electrode consisted of two polyurethane-coated copper wires (14 μm diameter Electrisola, Escholzmatt, Switzerland) as signal channels and a 50 μm diameter silver wire (Advent, Eynsham Oxon, UK). All three wires were twisted (two turns per mm) and had a length of 1 m each. One end of this bundle was soldered to a fitting DIP connector and attached to the head stages inside the hive box. The two copper wires were cut at the same length at the other end. The length of silver wire was 150 μm longer and bent perpendicular to the wire bundle at 250 μm distant from its tip. Thus the copper wires were free from the silver wire hook for 100 μm. Most of this length of the copper wires were inserted into the brain. The bent silver wire touched the brain surface and formed an electric connection, grounding the brain over the full length of the silver wire hook. The tips of the copper wires were plated with a gold and PEG8000 mixture as described in Ferguson et al. (2009). This treatment resulted in much lower input impedance at the head stage and was crucial for the length of the electrode keeping the noise at a minimum.

Once the experimental bee with the attached electrodes was moved on to the surface of the hive, the experiment started by connecting the electrode to a loose spring as described in Duer et al. (2015). This spring counterbalanced the weight of the electrode bundle and was designed such that it acted equally well across the comb surface. The spring was made of a fishing line (50 μm fishing line diameter, 6 cm diameter of the spool) carefully unspun from the spool by four turns. These four turns, when attached at one end to the box on top of the arena and unfolded to a very loose spring. The electrode bundle was attached at 1/5th of its length between the electrode holder and the spring. The remaining 4/5th of the electrode bundle elongated the spring by 20 cm, compensating the weight of the electrode of 8 mg.

The signals from the head stages were amplified (npi electronics, Tamm, Germany) and bandpass filtered from 10 Hz to 2 kHz. The signal was then sent through an active filter (Hum Bug, Digimeter, Hertfordshire, UK) filtering AC noise (50 Hz). This filter was only necessary during the dissection procedure when the shielded hood could not be lowered because the electrode was connected to the head stages inside of the hood. The neural signals were digitized at 40 kHz with an analog-to-digital-converter (1401 micro MKII, Cambridge Electronic Design, Cambridge, UK). The incoming data were inspected in real-time to determine the recording quality. The recording software (spike2, Cambridge Electronic Design, Cambridge, UK) introduced a digital bandpass filter from 300 Hz to 2 kHz. This software was used to save and synchronize the electrophysiological data with the video data containing the behavior. The raw spike traces were inspected off-line for a stable baseline to assure recording from the same unit. Once the criterion was met the spikes were sorted by semi-automated template matching in spike2. The resulting single units were tested in spike2 for homogeneous shapes within their template by principal component analysis. The units were tested whether they obey refraction time to indicate their single spiking source. If muscle potentials occurred, they were identified by their much slower timing and higher amplitude.

The videos (1,600 × 1,200 pixels, 10 frames per second) were analyzed by a custom made tracker. It extracted the coordinates and long body axis of the recorded bee as well as the closest other bees. Subsequently, the spikes were binned into 100 ms bins aligned with the behavioral data from the tracker. All further analyses and statistical tests were performed in MATLAB (MATLAB 2011, MathWorks Inc., Natick, MA, USA). Any data containing questionable spike traces or poor behavior were excluded. Poor behavior was determined visually and became apparent in the first few minutes. Bees running uninterruptedly in tight cycles or that did not move were excluded based on poor behavior.

Statistics: none of the data was normally distributed. The Wilcoxon rank-sum test was used for the following questions: (1) does the focal bees’ walking speed distribution differ from randomly selected bees’ walking speed distribution; (2) does the distributions of the distances to the closest bee differ between low and high spike rates; (3) do the differences in variance of spike rate distributions dependent on five social categories (“alone,” “random,” “walking onset,” “passive contact,” “active contact”); (4) does the phasic spike rate increase change the walking speed or the distance to the closest bee compared to random; (5) does the distance to the closest bee change the spike rate; and (6) do the differences in variance distributions of walking speed or distance to the closest bee depend on the spike rate increases during the time window of 4 s before. The Rayleigh test was used to test: (1) uniformly distributed spike rate distribution across orientation angle of the recorded honey bee in the relation of gravity; and (2) uniformly distributed spike rate distribution across the angle of the approaching bee and the long body axis of the recorded bee.

## Results

### Data Structure

**Figure 2:**
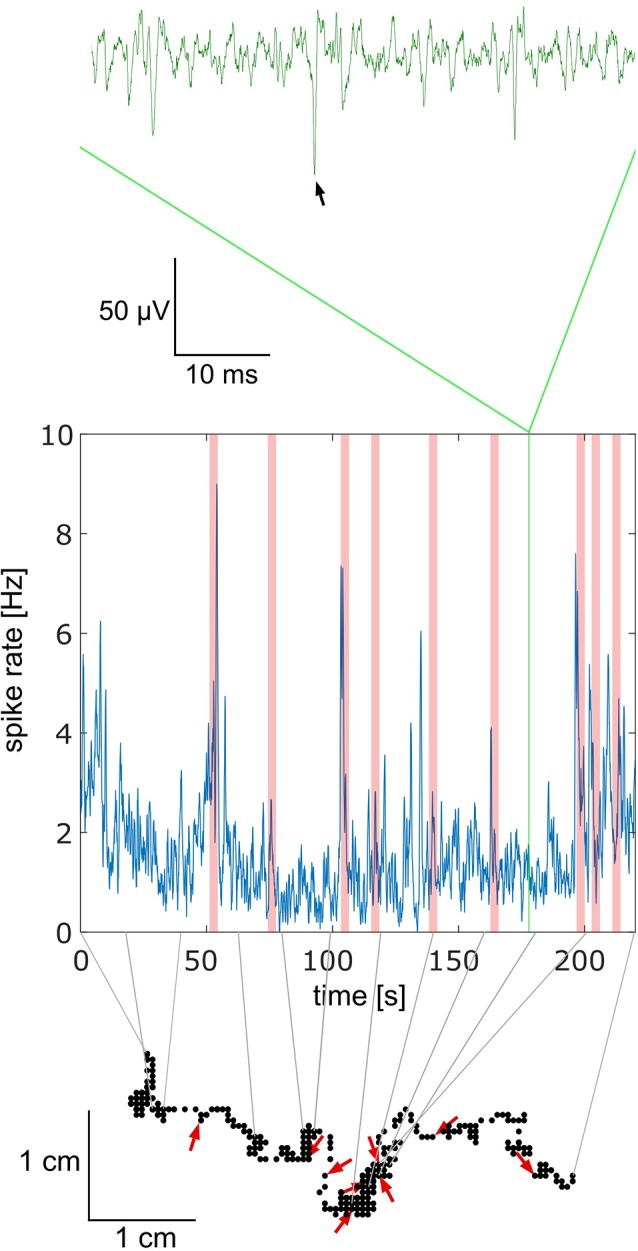
Relationship between behavior and electrophysiology. The upper trace in green shows a short part of the electrophysiological recording. Spikes were extracted using a semi-automated template matching in spike2 (see Supplementary Figure S1 for spike shape templates; the black arrow marks the selected spike). The frequencies of spikes over a 4 min period are shown in the graph below in blue. The green vertical bar in this graph indicates the time window of the raw green trace above. Light red vertical bars indicate social interactions, in this case, another bee approached the focal bee by coming closer than 1 cm. The lower part, made of black dots, shows the trajectory of the focal bee during the full-time window of recording shown in the middle graph. The gray lines connect time points between the spike rate (middle graph) and the trajectory (lower graph). Red arrows indicate the points of interaction between the focal bee and another bee. The direction of the red arrow indicates the approaching angle of the other bee. Note that no stimuli were given by the experimenter. Both the focal bee and any other bee were free in their behavior. The data shown come from bee A.

The data were collected during four spring/summer seasons. The animal from which the data came in a particular step of analysis will be called the focal bee. Due to the strict criteria, we applied for accepting data for further analyses the success rate was low (73 out of 800 bees). Most data were not further analyzed because of short or poor neuronal recordings. Out of the 73 experiments only 10 contained high-quality neuronal signals and close to the normal behavior of the focal bee. In all cases, only one unit was recorded. Spike shape plots of the 10 experiments are shown in Supplementary Figure S1. An exemplary short section of the walking trajectory together with the recorded spike activity is shown in Figure 2. Notice that no stimuli were given by the experimenter at any time during the whole experiment. Rather stimuli received by the focal bee were caused by itself or by another bee. Red arrows in Figure 2 mark close contacts with another bee at the focal bee’s trajectory and the corresponding moments in the spike rate trace with light red vertical bars.

The suitability of our basic experimental design required the proof that the recorded animal behaved similarly to the other bees in the small colony, and that the colony as a whole developed rather normally. Continuous video recordings (day and night) under weak infrared light of the experimental hive during experimental seasons were performed. These videos allowed us to observe the queen laying eggs, the queen group moving around with her, the feeding and development of the brood as well as the traffic at the hive exit and the waggle dancing of foragers. Based on these observations we confirmed the close to normal social life of the colony. During the experiment, the behavior of the recorded bee was video recorded and compared with that of the other colony members. The parameter used for behavioral comparison was the speed of walking. No significant differences were found for the 10 animals that were accepted for our analyses. The success rate of our experiments was very low indeed due to two conditions, the strict parameters applied to qualify close to the normal behavior of the recorded bee and the quality of the recordings. Seventy-three out of 800 preparations led to acceptable neural recordings during the first 10 min of the experiment, but only 10 of these 73 animals showed close to normal behavior and stable neural recordings. In most discarded recordings the animals showed no social behavior before the recording deteriorated.

### Walking, Body Direction and Place

First, we tested whether the focal bee showed normal walking behavior by comparing the walking speeds of all recorded bees with randomly selected bees (Supplementary Figure S2). The randomly selected bees were chosen from a pool of bees that were video tracked together with the recorded bee and were closest to the recorded bee to sample behavior as similar to that of the recorded bee as possible. No significantly different walking speed distributions were found (Wilcoxon rank-sum test; *p* = 0.95).

**Figure 3:**
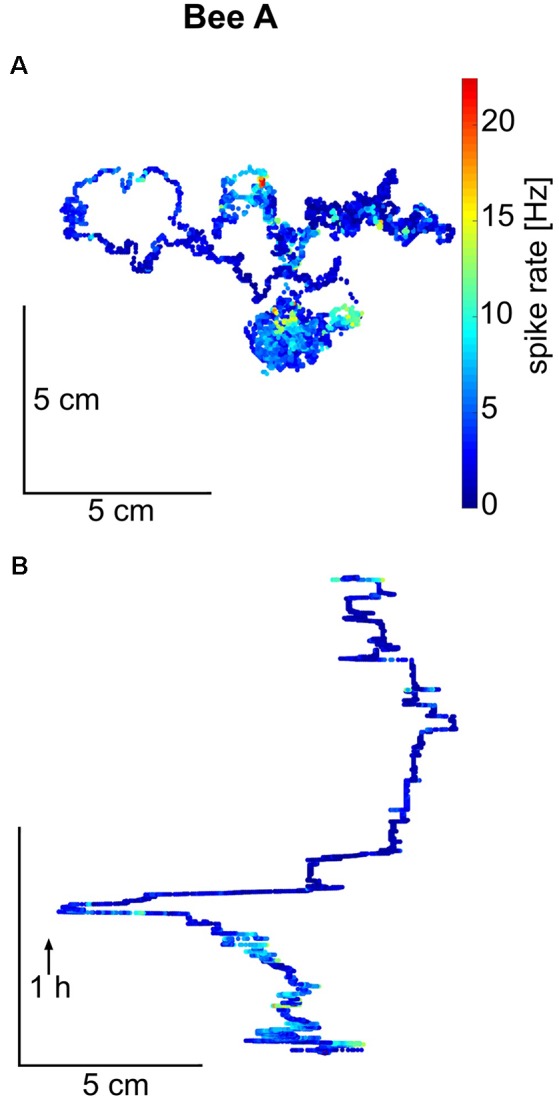
Trajectory and spike rate over time for honeybee A. **(A)** The total trajectory of bee A is plotted in two versions, as a projection on to the hive floor and in **(B)** as a trace over time for one spatial dimension (arrow indicates the direction of time). Spike rate (in 100 ms bins) is given in false colors on the trajectory. Bee A covered only a part of the hive floor and did not return to the same place multiple times from different directions. Similar walking trajectories are shown for the other bees in Supplementary Figure S3.

Next, we asked whether the units showed any place related activity changes. Figure 3 shows a representative example of the walking trajectory of a focal bee. Although there are places where the unit fired differently than at other places, this observation is limited to the fact that none of the focal bees covered the whole arena and rarely returned to the same location from multiple directions. These conditions are requirements for uncovering reliable place-related neural activity changes. The walking trajectories of all focal bees are shown in Supplementary Figure S3. By visually inspecting the trajectories and spike rates we conclude that none of the focal bees showed reliable place-related activity changes.

**Figure 4:**
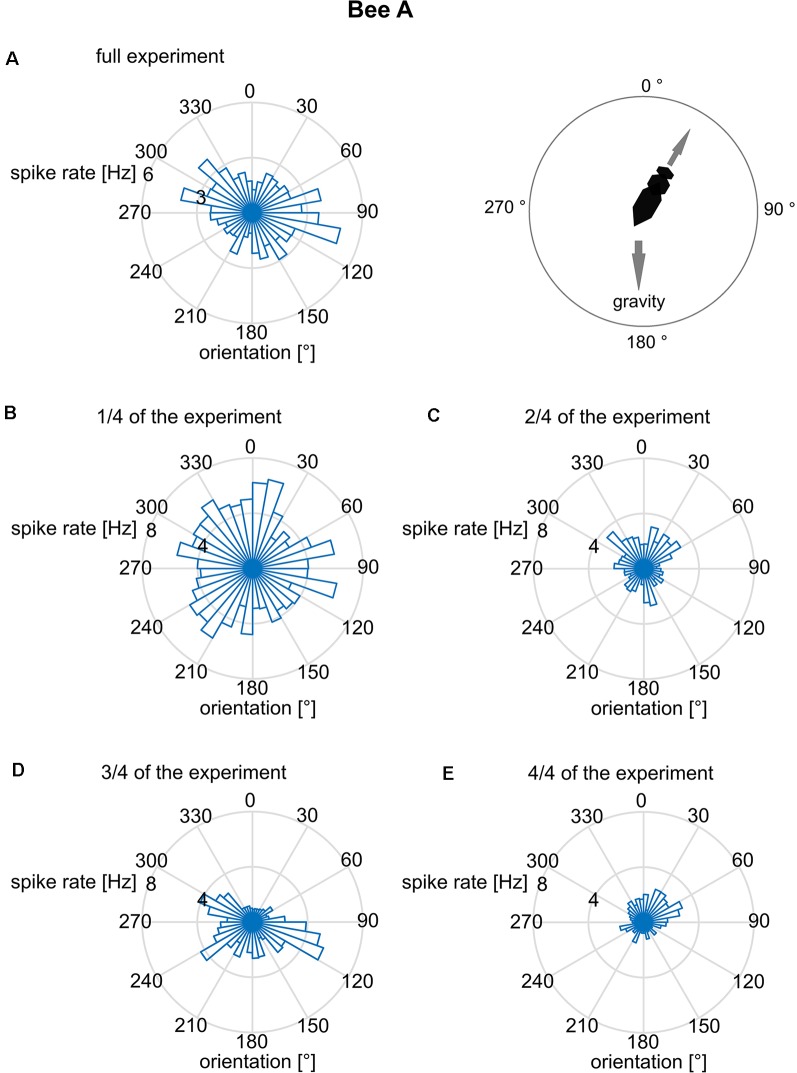
Mean spike rates in relation to the long body axis of bee A. The angles are given relative to gravity (180° = downwards). Spike rates are plotted for 10° bins of directions relative to gravity. The reliability of the relationship between spike rate and body direction was examined by plotting not only the full data set **(A)** but also sub-datasets [**B**: first quarter of data, **(C)**: second quarter, **(D)**: third quarter, **(E)**: fourth quarter]. Although the full data set indicated a bimodal distribution, no stability over time was found. The same analysis was performed for the other bees B–J and no time constant directional effect was seen.

We further addressed the question of whether the orientation of the focal bee in relation to the slope of the arena was correlated with the spike rate. Such a body directional effect could result from the focal bee’s orientation to gravity since the base of the hive box was tilted by 17°, and bees are known to detect slopes of >10° (Markl, 1974). The bee may also orient to the light shining through the exit hole. When inspecting the related polar plot (Figure 4), none of these spike rate data was randomly distributed (Rayleigh test, all *p* < 0.001). This applies to both the whole experimental period (Figure 4A) and the data cut into quarters of time sections (Figures 4B–D). A bimodal directional distribution of spike rates was found in some of the time sections (see Figures 4B–D). However, this effect is unstable and changes over time making it unlikely to be a reliable effect. Thus, just like bee A, all other bees spike rate distribution effects did not mutually confirm each other.

### Distance Between the Focal Bee and the Closest Next Bee

**Figure 5:**
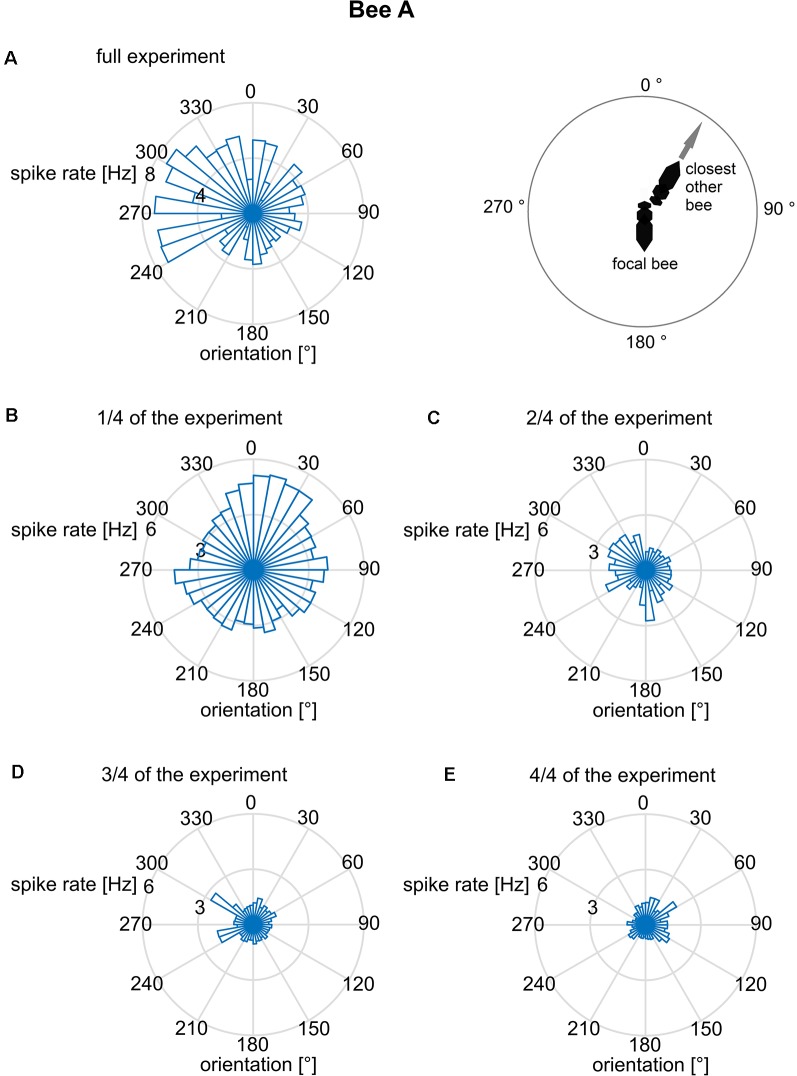
Mean spike rates in relation to the directions of the long body axes of the focal bee A and that of the closest other bee. Spike rates were averaged for 10° directional bins. The reliability of the relationship between spike rate and body direction was examined by plotting not only the full data set **(A)** but also sub-datasets [**B**: first quarter of data, **(C)**: second quarter, **(D)**: third quarter, **(E)**: fourth quarter]. Although the full data set indicated a preferred direction to the left side of the focal bee, no stability over time was found. The same analysis was performed for the other bees B–J and no directional effect was found.

**Figure 6:**
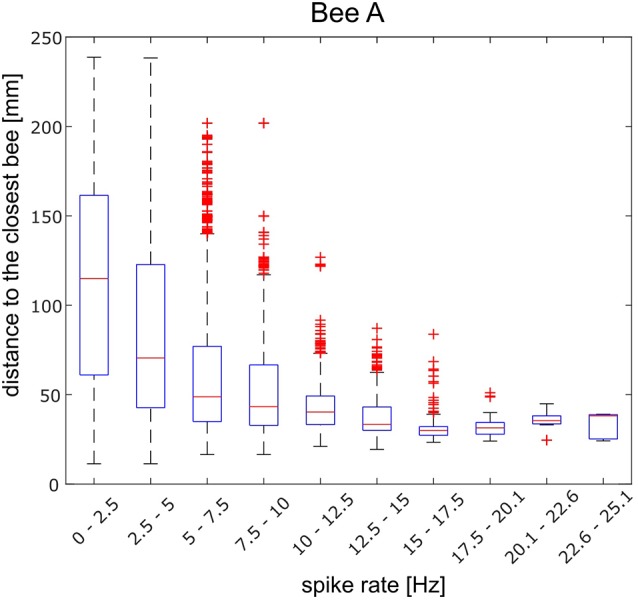
Distance between the focal bee A and the closest next bee and spike rate calculated for 10 equal-sized frequency groups (*abscissa*). The highest 10% spike rate bin appeared for the shortest distance and was different from the distance distribution of the lowest spike rate bin (Wilcoxon rank-sum test; *p* < 0.001). Similar calculations for the other bees are shown in Supplementary Figure S4.

Social contacts in the dark hive are most likely initiated by odor stimuli. We, therefore, analyzed the spike activity changes during times in which another bee came close to the focal bee. Since antennal stimulation and even contacts might be particularly important we sorted spike rate changes in relation to the distance and direction with the closest other bees. The angle of the long body axis of the focal bee and the long body axis of the closest bee showed no reliable effect on the spike rate distribution as they were not stable over time (Figure 5). Also, we examined the data for each quarter for an effect of distance to the next closest bee by dividing it into occasions where the distances were either below or more than 5 cm. Again no stable effect over time was found. Low spike rates could occur at moments when other bees were close as well as when they were far away. Higher spike rates, however, appeared only when the closest bee was close to the focal bee (Figure 6).

Next, we analyzed the effect of distance to the closest bee in more detail. The spike rate changes showed a wide bandwidth. Spike frequency ranged from well below 0.1 Hz (average minimum of 0.63 Hz) to well above 40 Hz (average maximum 23.3 Hz). Spike frequencies were divided into 10 part and the corresponding distances were plotted as boxplots (Figure 6). The distances observed during highest 10% spike rates were significantly different from the distances observed during the lowest 10% spike rate in all 10 animals besides bee B and bee H (Wilcoxon rank-sum test; bee A: *p* < 0.001, bee B: *p* = 0.26, bee C: *p* < 0.001, bee D: *p* < 0.001, bee E: *p* < 0.001, bee F: *p* < 0.001, bee G: *p* < 0.001, bee H: *p* = 0.11, bee I: *p* < 0.001, bee J: *p* < 0.001). In some animals, high frequencies occurred at closest distances (bees A, D, I) and low spike rates at any distance. A low spike rate was, therefore, not informative for the distance between the focal bee and the closest bee. However, a high spike rate was. Figure 6 shows the results for bee A as an example. In other animals (bees C, J) the distance and spike rate relation was inverted, high spike rates were informative for the animal being alone and low spike rates occurred at any distance. The results for all other recorded bees are given in Supplementary Figure S4.

### Social Contacts

**Figure 7:**
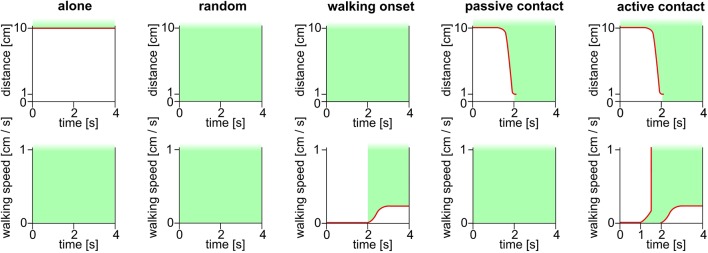
Schematic depiction of the definitions of social states. The critical parameters were the distance between the focal bee and the next other bee (upper row, ordinate) and the walking behavior (lower row). State “alone” was defined by the focal bee not having any other bee closer than 10 cm for 4 s independent of the walking speed of the focal bee. The state “random” was not a social state in the biological sense but an important control to determine baseline values. The “random” states were selected randomly by software. The state “walking onset” was defined by the focal bee moving less than 1 mm for 2 s and then moving continuously for at least 2 s. The walking onset was synchronized to the 2 s mark of the 4 s window. This state was independent of the distance to the closest other bee. The “passive contact” state was defined by being alone in the beginning and having an encounter with another bee at the 2 s mark of the 4 s window. At the beginning of the window, the focal bee had no other bee closer than 10 cm. At the 2 s mark another bee, only one bee, must have been at least 1 cm close to the focal bee. The “passive contact” state only applied if the focal bee did not move during the entire 4 s window, or the focal bee moved for the entire 4 s window. For this state movement as defined for “walking onset” did not occur. In contrast, for the state “active contact” the focal bee was initially alone (no other bee closer than 10 cm) and then one other bee came closer than 1 cm to the focal bee. This moment was aligned to the 2 s mark of the 4 s window. The state “active contact” was applied if the focal bee was standing still in the beginning and started moving between the 1 s mark and the 2 s mark of the 4 s window. All detected time windows lasted 4 s. Green areas depict periods and values that are critical for the respective definition. Red lines highlight thresholds.

We have observed a relationship between the recording of high spike rates and the distance between the focal bee and the closest other bee. To uncover relations between social contacts and the spike rate we defined social states. From the parameters walking speed of the focal bee and distance to the closet next bee, we came up with 4 social states: “alone,” “walking onset,” “passive contact” and “active contact,” and the additional state “random” (Figure 7). A time window of 4 s centered on the moment of first contact or first movement was chosen. A similar time windows was randomly collected for comparison (condition “random”). The social state “alone” was defined by a 4 s window in which the focal bee had no other bee close by (e.g., no other bee was closer than 10 cm to the focal bee). The state “walking onset” was defined as the focal bee not moving for 2 s and then moving for at least 2 s continuously. The states “passive contact” and “active contact” were defined as time windows in which the focal bee was alone beforehand and then only one bee came closer than 1 cm. The 2 s mark of the 4 s time window was synchronized to the moment when the two bees got into contact. “Passive contacts” included only such events during which the focal bee did not change its walking behavior, e.g., the focal bee walked the whole time window or did not move the entire time. In contrast, “active contact” included only contacts where the focal bee started walking between 1 s before the contact or later up to the moment of contact.

**Figure 8:**
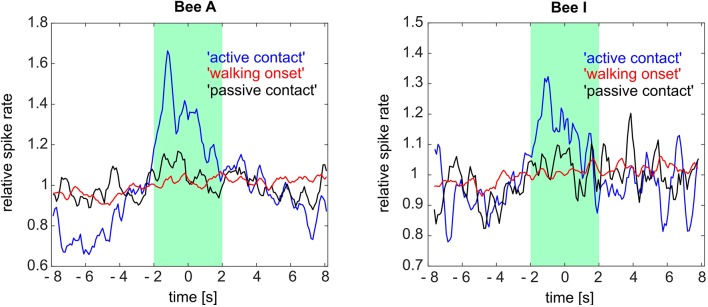
Perievent time histogram (PETH) for three social states (“active contact,” “walking onset,” “passive contact”) of two focal bees (bee A and bee I). Averaged relative spike rates for all these social states are plotted on the ordinate. The three social states were temporally aligned with the moment of contact or walking onset, respectively (time point 0 on the *abscissa*). The “active contact” spike rate of bee A as well as bee I showed a phasic spike rate increase 1.2 s (bee A) and 1.1 s (bee I) before the “active contact.” The other two social states did not show any tendency of change in their related spike rates within the same time window (8 s before and 8 s after the event onsets). Green box: 4 s social state windows synchronized with the time point 0. See Figure 7 for the definition of the social states and Figure 9 for further results.

To test whether the 4 s time window is appropriate we calculated the perievent time histograms (PETH) over an extended time window (8 s before and 8 s after state) for three of the social states (“walking onset,” “passive contact” and “active contact”). PETHs were calculated to quantify the temporally resolved spike rate changes (Figure 8). We found in many but not all cases that the phasic spike rate increased before the contact was reached. These phasic spike rate changes were particularly prominent in bees A and I for the behavior “active contact.” No such changes were found for “passive contact” and “walking onset.” It is thus unlikely that odor stimuli emanating from another bee close by may have caused the neural effect, rather it reflects a neural process connected to the decision of becoming actively engaged in a contact with another bee. We conclude, therefore, some of the ENs predicted a future behavioral act and thus may have been involved in a neural decision-making process.

So far we quantified the neural events by correlating spike rate changes to behavioral events. Applying such a strategy we assumed a selective role of the recorded neuron in the neural processes involved in the sensory and higher-order integrative processes. ENs, however, are not selective, neither in the processing of sensory information nor in the control of cognitive tasks. Multimodality, experience-dependent plasticity, memory processing and retrieval, novelty detection, attention dependence, and other processes have been described for MB ENs (Filla and Menzel, 2015) rather similar to what is known about neurons of the prefrontal lobe of mammals (Davis et al., 2019). Thus ENs are most likely also involved in several or multiple functionally clusters of neurons serving partially overlapping cognitive functions as e.g., prefrontal neurons are (Mante et al., 2013; Rigotti et al., 2013). Information transfer of neurons potentially participating in several neural clusters has been related to their higher fluctuations in spike activity due to multiple overlapping excitatory and inhibitory inputs (Salinas and Sejnowski, 2001). Spike rate variance may thus be an indication for higher-order information processing. We, therefore, looked into spike rate variance as a measure of the coding property of the recorded neurons.

Indeed, when visually inspecting the spike rate in 4 s windows of the “alone” condition we observed less spike rate variance as compared to “active contact” where the spike rate often increases or decreases at different time points within this 4 s window (Figure 8). The amplitude and direction of spike rate changes varied as well. Therefore, we captured these changes by quantifying the spike rate (in 100 ms bins) variance as the average of the squared differences from the mean. All spike rates within the 4 s window were computed into this single parameter of variance leading to a reduction of complexity, independent of the mean spike rate and the polarity of the rate change. As pointed out above we performed this analysis separately for the different animals because most likely we recorded from different units in different animals.

**Figure 9:**
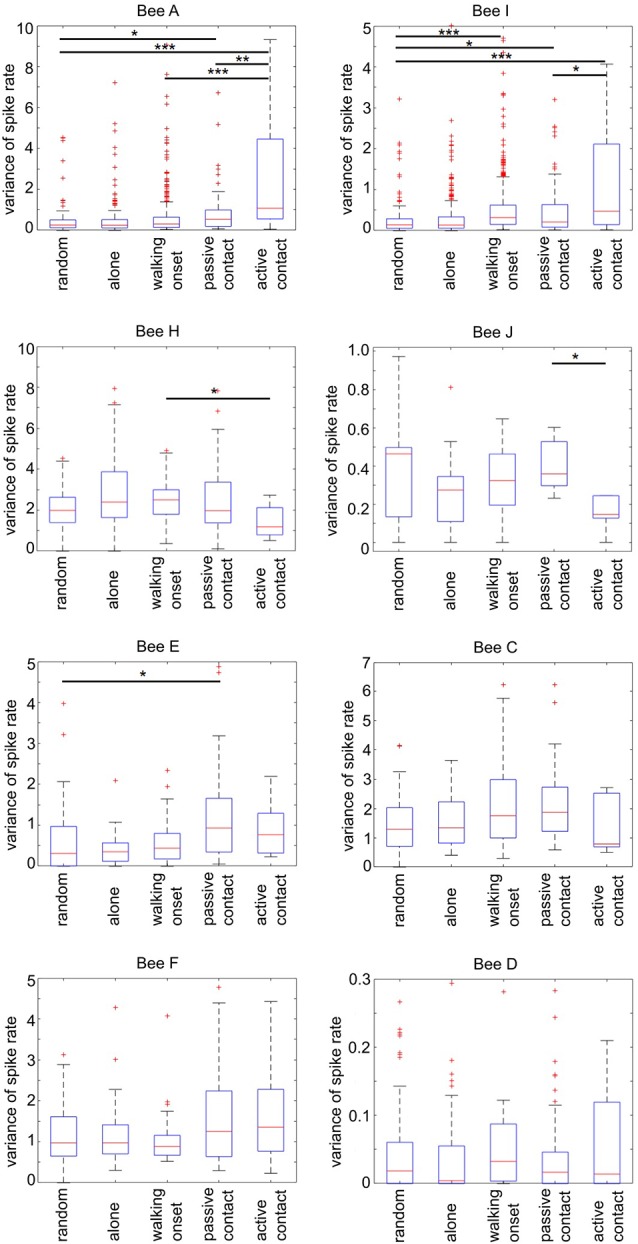
Spike rate variance during different social states. Each graph plots separately for each focal bee the parameter of spike rate variance (ordinate) during a 4 s time window defined by five social states (for details see Figure 7). The number of “random” states was chosen to be equal to each of the naturally occurring “passive contact” states. Statistics (Wilcoxon rank-sum test). The horizontal lines mark the statistically significant differences in spike rate variance (Bonferroni corrected: alpha/6; one star: *p* ≤ 0.0083, two stars: *p* ≤ 0.0017, three stars: *p* ≤ 0.00083, Wilcoxon rank-sum test). Spike rate variance was significantly different in four animals out of eight between passive and active contact (focal bees A, I, H, J). Notice that the direction of difference was either higher variance in active contact (bees A and I) or lower (in bees H and J).

Figure 9 shows spike rate variance during different social states in all experimental animals besides bee B and G which did not experience any social interactions (all variance distribution differences were tested *via* Wilcoxon rank-sum test). Spike rate variance was significantly higher than “random” and “passive contacts” during active contacts in bees A and I (first row). Also, both bees showed higher variance during “walking onset.” The animals H and J given in the second row of Figure 8 were characterized by opposing effects compared to animals A and I. The variance of spike activity during “active contact” was lowest compared to other states. Furthermore, bees H and J showed significant differences between the “passive contact” and the “active contact” states. Four remaining bees (E, C, F, D) given in the third and fourth rows of Figure 9 show only two significantly different spike rate variances (bee E: “passive contact” vs. “random,” bee C: “walking onset” vs. “random”).

In summary, the variance of spike rate was high in bees A and I and low in bees H and J when the focal bees showed “active contact” behavior. Both effects were selective for “active contact” since the variance of spike rate was significantly different from “passive contact” and “walking onset.” These different forms of correlation of spike rate variance with states of social interaction suggest neuron-specific mechanisms potentially involved in the neural control of social behavior.

## Discussion

Neural correlates of social behavior are expected at a high level of neural integration since multiple sensory inputs from both the outer and the inner world need to be interrelated with the social role of the individual animal in focus and the conditions of the whole society. We expect this level of high neural integration in MB ENs based on the global wiring pattern of the honeybee brain (Brandt et al., 2005; Rybak et al., 2016) and the results of multiple intra- and extracellular recordings from MB ENs (review: Menzel, 2012). MB ENs are not tuned to any selective sensory modality or motor pattern rather their response characteristics include multiple forms of highly processed sensory information across modality, experience-dependent plasticity and attentional states (review: Menzel, 2014). We did not expect single ENs to be selective for any particular social state. Rather groups of ENs are likely to interact by forming functionally overlapping clusters of neurons involved in social states. Here we recorded extracellularly from MB ENs belonging to the A1, A2 and A5 groups (Rybak and Menzel, 1993) and we found some evidence for neural activity patterns linked to self-induced social interactions.

The goal of our experimental design was to search for neural correlates of undisturbed and freely running social interactions. The recorded neurons needed to be analyzed separately because only one unit was recorded in each animal and most likely different neurons were recorded in different animals. Therefore, our analyses were based on comparisons in which we aligned spike activity to the particular behavior in question. Such a strategy of data analysis was also necessary because no stimuli were given to the recorded animal and no manipulations of the social conditions were performed. Multiple stimuli will have reached the focal bee particularly when other bees approached her or when she moved towards another bee or when she moved into areas of the colony characterized by different social environments (queen group, food store, exit of the hive). No area-specific neural activities were found excluding the possibility that the recorded MB ENs encode areas of social environments or locations within the colony. We also did not find any place-related neural activities (Figure 3) and no body direction related spike activities (Figure 4) excluding the possibility that they were involved in spatial navigation inside the hive. This finding differs from those of Mizunami et al. (1998) who reported place-related activity patterns of MB ENs in cockroaches trained to a heated place. However, we cannot exclude the possibility that particular combinations of conditions (e.g., body directions at particular places) might correlate with spike rate changes. Walking behavior *per se* was also not correlated with spike rate differences excluding a role in premotor control. This is different from what has been observed in ENs in cockroaches that were found to increase the spike rate with the initialization of locomotion (Okada et al., 1999).

Searching for spike rate correlations with social interactions we selected a 4 s time window by inspecting any spike rate changes before, during and after social interactions in ranges of hundreds of milliseconds up to a few seconds. The highest spike frequencies occurred when the focal bee was close to another bee. From the 10 tested bees, eight showed a significant difference when comparing the highest 10% of spike rate with the lowest 10% during encounters with the closest other-bee. When the units fired at a low-frequency other bees may have been close or not but when the frequency of spikes was high another bee was in some animals always close (Figure 6). The direction of the contact to another bee, however, did not affect spike rate (Figure 5) indicating a more global odor effect of a close-by bee rather than a specific and spatially directed olfactory stimulation effect. A phasic increase of spike rate was found to precede active contacts in two out of 10 bees (Figure 8). Taken together an active contribution of the focal bee appears to be an essential component of spike rate changes.

Most interestingly spike rate variance increased, decreased or did not change during active social contacts depending on the particular neuron (Figure 9). As pointed out above coding properties of ENs are likely to be realized in functionally overlapping clusters. Such clusters are most likely characterized by multiple excitatory and inhibitory interactions leading to the increased variance of spiking in single neurons. Assuming rate based information processing, low variance in spike rate might relate to a stable firing rate and may, therefore, transmit only low information flow through the unit. The higher the variance of spike rate for a given time window, the more information may be processed. This somewhat speculative argument is supported by the finding that recurrent networks transiently modulate neural excitability leading to higher “noise” (also called “dark activity”) in single cortical neurons (Davis et al., 2019). “Dark activity” is a useful term in this context because it is unknown how fluctuations in spontaneous neural activity at the single neuron level are created in recurrent networks although it is clear that these fluctuations play a key role in shaping the responsiveness to incoming signals, reflect the state of the intrinsically modulated network and enhance information transfer between populations of neurons (Engel et al., 2001; Salinas and Sejnowski, 2001). The MB in the honeybee is characterized by prominent recurrent neurons (Zwaka et al., 2019) whose neural activity relates to novelty detection, context-dependence, and expectation (Filla and Menzel, 2015).

MB ENs in more than half of the animals (six out of eight bees, Figure 9) showed a significantly different spike rate variance when the focal bee was in some form of an active state as compared to similar time windows when the focal bee was alone or when the time windows were randomly selected. In four of the eight animals, the spike rate variance was significantly higher in the active contact state (Figure 9) indicating the importance of an initiative or an active probing state as a component in the units “dark activity.” Particularly strong differences in spike rate variance were found in bees A and I during active contacts as compared to randomly selected time windows, walking onset without leading to social contact and passive contacts. The units recorded in bee A and I did also not change their firing rates and their spontaneous firing variance when the bees received stimulation by a hive mate. Bee I differed from bee A in the sense that it’s MB EN showed a statistically significant increase in spike activity when the bee started walking as compared to similar randomly selected time windows. The MB ENs of bees J and H showed a lower variance of firing rate leading to significantly different variance in bee J only during “active contacts” as compared to “passive contact” and “walking onset.” Changes in spike rate variance were not found in MB ENs of the bees D and F. These results indicate that the recorded MB ENs differed in their involvements in circuits likely to be related to active social contacts.

Based on our hypothesis we conclude that increased spike rate variance and the strong specificity predicting active social contacts (as in bees A and I, Figures 8, 9) may reflect a higher involvement in neural clusters controlling active social contacts possibly including stronger inputs from recurrent neurons. Other ENs as in bees J and H characterized by reduced changes in spike rate variance and less specificity may be more likely to be less involved, and those recorded in bees D and F possibly not involved at all.

We recorded MB ENs in honeybees acting freely in their natural environment of the social community. The bees were free to participate in the social life and did so as the other bees. No particular stimuli were applied. Neural activities were closely related to social interactions. The most specific effect was found during self-initiated walking towards a hive mate. Spike rate variance correlated with specific states of social behavior. We hypothesize that spike rate variance possibly reflects hidden neural activities (“dark activity”) characteristic for neural circuits with excitatory and inhibitory recurrent connections. Recordings from recurrent inhibitory MB ENs (A3 neurons) will be particularly interesting in exploring their involvement in controlling social interactions.

## Data Availability Statement

The datasets of all 10 bees for this study can be found here: https://figshare.com/articles/Neural_correlates_of_social_behavior_in_mushroom_body_extrinsic_neurons_of_the_honeybee_Apis_mellifera/11341817/1 and https://doi.org/10.6084/m9.figshare.11341817.v1. The MatLab code used for analysis can be found here: https://github.com/Neuro3en/NeuronalCorrelatesSocialSignalsHoneyBees.

## Author Contributions

RM and BP conceived the aim and the design of the study, wrote sections of the manuscript and finalized the manuscript. IF, AD, IH, and ID were involved in conducting the experiments. BP performed the statistical analysis and wrote the first draft of the manuscript. All authors contributed to manuscript revision, read and approved the submitted version.

## Conflict of Interest

The authors declare that the research was conducted in the absence of any commercial or financial relationships that could be construed as a potential conflict of interest.
