# Supplementary material for: Neural Correlates of Social Behavior in Mushroom Body Extrinsic Neurons of the Honeybee Apis mellifera
Source: Front Behav Neurosci. 2020 Apr 21;14:62. doi: 10.3389/fnbeh.2020.00062 (PMC7186758; doi:10.3389/fnbeh.2020.00062)
Supplement: Supplementary file 1 [file Data_Sheet_1.PDF]

Supplementary Material

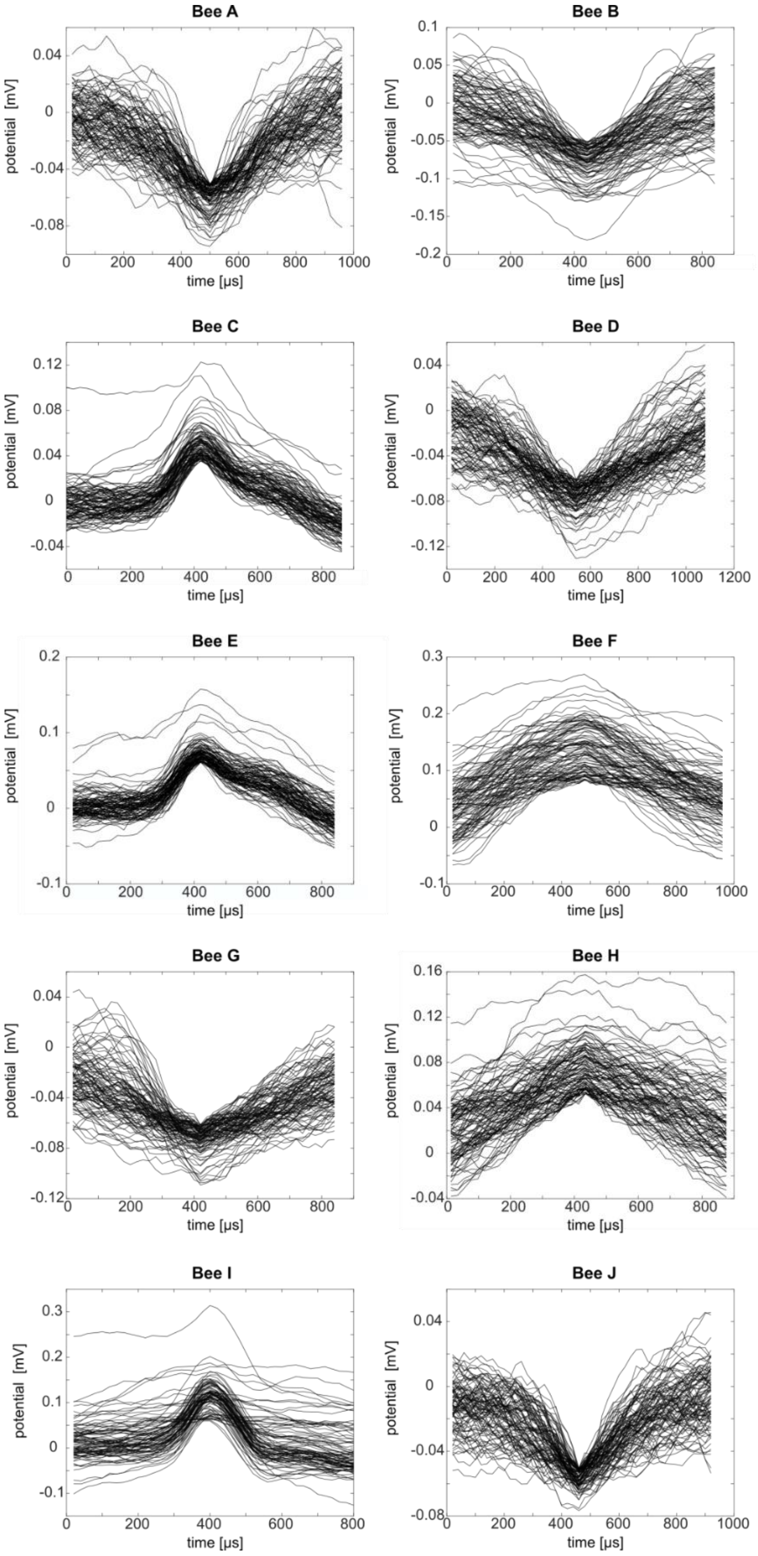

Supplementary Figure 1. Spike shape analysis. Each graph shows 100 randomly selected spikes from the electrophysiological recordings of experimental bees A – J. The spikes were extracted using a semi-automated template matching procedure as implemented in spike2 software. The uniformity of the respective spikes was confirmed applying principal component analysis as an indicator of one single neuronal source.

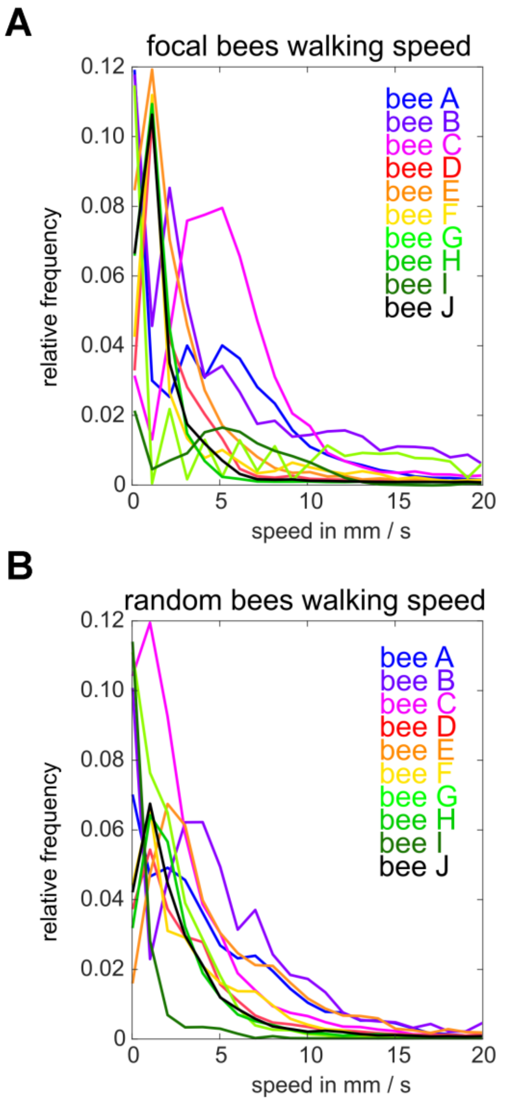

Supplementary Figure 2. Comparison of the walking speed of focal bees and randomly selected other bees. The walking speed was measured by the changes of coordinates of the bee body (center of mass) with a temporal resolution of 100 ms. (**A**) Distribution of walking speeds for each focal bee. (**B**) Frequency of walking speed of the closest other bee to the respective focal bee A -J. The inset gives the respective focal bee. The closest bee was chosen because there may be local differences on the ground floor possibly influencing walking speed. The data for the focal bee and its nearest other bee were selected at the same time of day. The same number of data were collected for the focal bee and its closest other bee. The pooled average walking speed for each pair of bees, focal and other bee, was compared (Wilcoxon rank-sum test) and no differences were found ($p \geq 0.95$).

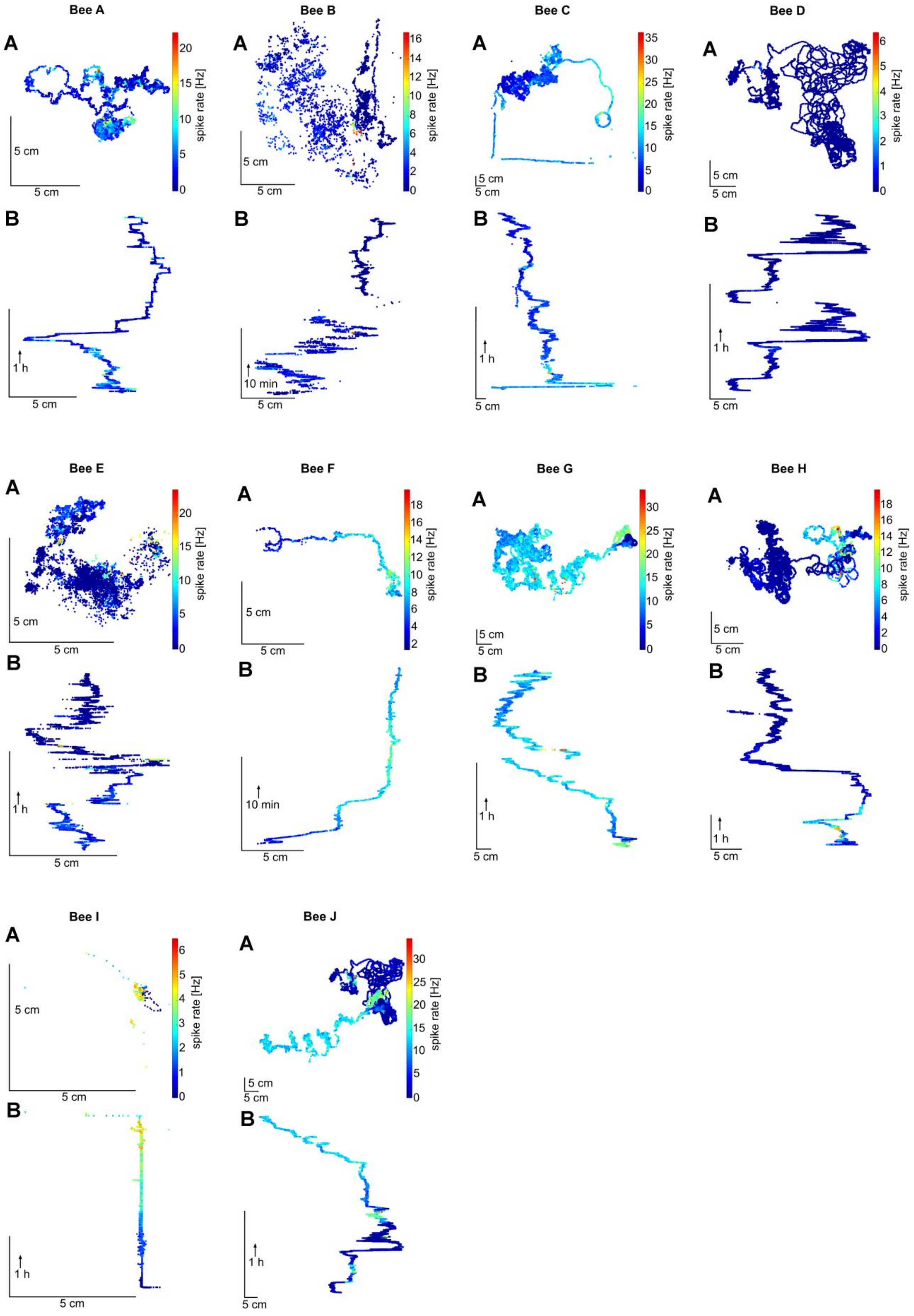
Bee A
A
5 cm
5 cm
spike rate [Hz]
0
5
10
15
20
B
1 h
5 cm
Bee B
A
5 cm
5 cm
spike rate [Hz]
0
2
4
6
8
10
12
14
16
B
10 min
5 cm
Bee C
A
5 cm
5 cm
spike rate [Hz]
0
5
10
15
20
25
30
35
B
1 h
5 cm
Bee D
A
5 cm
5 cm
spike rate [Hz]
0
1
2
3
4
5
6
B
1 h
5 cm
Bee E
A
5 cm
5 cm
spike rate [Hz]
0
5
10
15
20
B
1 h
5 cm
Bee F
A
5 cm
5 cm
spike rate [Hz]
2
4
6
8
10
12
14
16
18
B
10 min
5 cm
Bee G
A
5 cm
5 cm
spike rate [Hz]
0
5
10
15
20
25
30
B
1 h
5 cm
Bee H
A
5 cm
5 cm
spike rate [Hz]
0
2
4
6
8
10
12
14
16
18
B
1 h
5 cm
Bee I
A
5 cm
5 cm
spike rate [Hz]
0
1
2
3
4
5
6
B
1 h
5 cm
Bee J
A
5 cm
5 cm
spike rate [Hz]
0
5
10
15
20
25
30
B
1 h
5 cm

Supplementary Figure 3. Trajectories and spike rates over time for focal bees A - J. (**A**) The whole walking trajectory of the respective bee plotted together with the spike rate (time frame:100 ms) in false color. Notice that the color scale may be different for different focal bees. (**B**) The x – coordinate of the respective bee's trajectory on the abscissa and the time of recording on the ordinate together with the spike rate in false color. The spike rate to color conversion is given in each sub graph **A**. Bee C walked around the food container (middle right) and walked along the border at the lower and left edge.

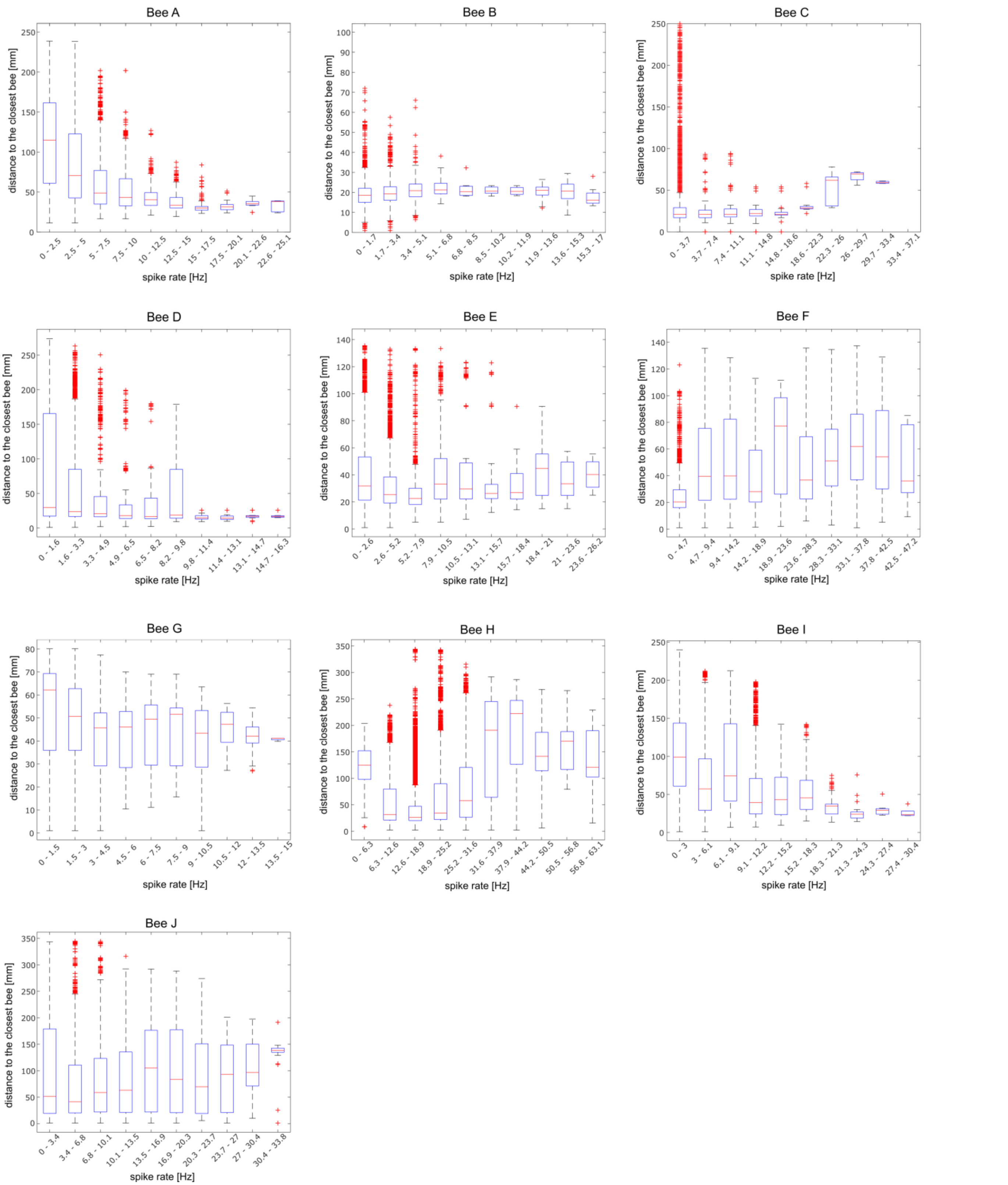
Bee A
Bee B
Bee C
Bee D
Bee E
Bee F
Bee G
Bee H
Bee I
Bee J
distance to the closest bee [mm]
spike rate [Hz]

Supplementary Figure 4. Distributions of distances to the closest other bee in relation to spike rate of the focal bee. The highest and lowest spike rates that occurred during each experiment defined the ranges in which 10 % proportions were segregated as given on the abscissa. The highest and the lowest 10% spike rate bin were significantly different in the following cases: bee A: $p < 0.001$, bee C: $p < 0.001$, bee D: $p < 0.001$, bee E: $p < 0.001$, bee F: $p < 0.001$, bee G: $p < 0.001$, bee I: $p < 0.001$, bee J: $p < 0.001$). Bee B ($p = 0.26$) and bee H ($p = 0.11$) did not show significant differences (Wilcoxon rank-sum test).
